# Gender-Based Differences in Aneurysm Characteristics Among Patients With Aneurysmal Subarachnoid Hemorrhage: A Multidetector CT Angiography Study at a Tertiary Care Hospital

**DOI:** 10.7759/cureus.84162

**Published:** 2025-05-15

**Authors:** Anshuman Tripathy, Mamata Singh, Pradosh Kumar Sarangi, Sasmita Parida, Sudhansu Sekhar Mishra

**Affiliations:** 1 Radiodiagnosis, Acharya Harihar Post Graduate Institute of Cancer, Cuttack, IND; 2 Radiodiagnosis, Jajati Keshari Medical College and Hospital, Jajpur, IND; 3 Radiodiagnosis, All India Institute of Medical Sciences, Deoghar, Deoghar, IND; 4 Radiodiagnosis, Padmini Care Hospital, Tangi, IND; 5 Neurosurgery, Institute of Medical Sciences (IMS) and SUM Hospital (Campus 2), Bhubaneswar, IND

**Keywords:** aneurysm, aneurysmal subarachnoid hemorrhage (asah), anterior cerebral artery (aca), ct head angiography, internal carotid artery (ica), multidetector computed tomography (mdct), subarachnoid hemorrhage

## Abstract

Background: Aneurysmal subarachnoid hemorrhage (aSAH) is a life-threatening neurovascular emergency. Gender-related variations in aneurysm characteristics may influence clinical presentation, imaging findings, and management strategies. This study aimed to evaluate the frequency distribution and gender-based differences in aneurysm characteristics among patients with aSAH using multidetector computed tomography (MDCT) angiography.

Methods: This cross-sectional observational study was conducted in the Department of Radiodiagnosis, Sriram Chandra Bhanj Medical College and Hospital (SCBMCH), Cuttack, a tertiary care hospital in the eastern region of India. A total of 111 patients with MDCT angiography-confirmed aSAH were included. Aneurysm features such as size, shape, multiplicity, and location were assessed. Statistical analysis was performed using the Chi-squared test, Fisher's exact test, t-test, and Z-test, with a p-value of <0.05 considered statistically significant.

Results: Of the 111 patients, 73 were female and 38 male (female-to-male ratio: 1.9:1). Female patients were significantly older than male patients (mean age: 56.11 versus 49.95 years; p = 0.020). Multiple aneurysms were more common in female patients (19.18%) than in male patients (5.3%; p = 0.0518). Aneurysms < 6 mm were more frequent in female patients (66.67%), while larger aneurysms (>10 mm) were more prevalent in male patients (20%; p = 0.0285). Fusiform aneurysms were significantly more common in male patients (15% versus 3.5%; p = 0.0273). Anterior circulation was involved in 92.22% of cases. Internal carotid artery (ICA) aneurysms predominated in female patients (48.27%), whereas anterior cerebral artery (ACA) aneurysms were more common in male patients (42.5%; p = 0.035).

Conclusion: Gender-based differences in aneurysm characteristics, including age at presentation, size, shape, and location, were evident in patients with aSAH. Awareness of these variations is essential for accurate diagnosis, individualized risk assessment, and development of tailored management strategies.

## Introduction

Aneurysmal subarachnoid hemorrhage (aSAH) is a fatal cerebrovascular disorder that is caused by bleeding into the subarachnoid space due to the rupture of an intracranial aneurysm. Although it accounts for only 5%-10% of all strokes, it is associated with significantly high rates of morbidity and mortality. Early diagnosis and accurate characterization of aneurysms are necessary for optimization of management strategies and improvement of patient outcomes [1].

The morphology and distribution of cerebral aneurysms can be variable based on several factors, such as age, gender, and other demographic characteristics. A previous study has found a higher incidence of aSAH in female patients, particularly post-menopausal women, likely due to hormonal influence on aneurysm formation and rupture risk [2]. However, recent studies have found no statistically significant difference in prevalence in women [3,4]. Furthermore, aneurysm features such as size, shape, multiplicity, and location significantly influence the likelihood of rupture and clinical presentation [3].

Multidetector computed tomography (MDCT) combined with CT angiography (CTA) has been the cornerstone in the non-invasive evaluation of aSAH. It offers high-resolution images enabling precise detection, localization, and characterization of intracranial aneurysms, thus supporting accurate diagnosis and effective treatment planning. Although digital subtraction angiography (DSA) is traditionally considered the gold standard for aneurysm detection, it is expensive and invasive and carries a risk of permanent neurological complications [5]. In contrast, CTA is cost-effective and safe and can be performed immediately following a routine non-contrast CT (NCCT) for rapid assessment [6,7].

Despite all the advancements in imaging techniques, there is limited region-specific data on gender-based variations in aneurysm characteristics among aSAH patients, particularly within the Indian population. Therefore, this study aims to analyze the frequency distribution and gender-related differences in aneurysm characteristics among patients with aSAH using MDCT angiography with a focus on patient demographics and aneurysm morphology, including size, shape, location, and multiplicity.

## Materials and methods

Type and settings

This hospital-based cross-sectional observational study was conducted in the Department of Radiodiagnosis at Sriram Chandra Bhanj Medical College and Hospital (SCBMCH), Cuttack, Odisha, a tertiary care hospital in the eastern region of India. The study period spanned from November 2020 to October 2022. The target population comprised patients diagnosed with aneurysmal subarachnoid hemorrhage (aSAH) based on CT angiography (CTA) findings who were admitted to our hospital during the study period.

Patients were included in the study if they had evidence of aneurysmal subarachnoid hemorrhage (aSAH) on CT angiography and were aged 18 years or above. Exclusion criteria were as follows: (1) history of trauma, (2) presence of ischemic stroke, (3) frank intraparenchymal hemorrhage, (4) absence of aneurysm on CTA, and (5) incomplete clinical or imaging data or refusal to provide informed consent.

Ethical consideration

Ethical approval for the study was obtained from the Institutional Ethics Committee of SCBMCH, Cuttack, Odisha (IEC number: 423/2020). Informed consent was secured from all participants before enrolment and imaging. For patients who were unconscious or unable to provide consent, permission was obtained from their next of kin or legal guardian in accordance with ethical guidelines.

Imaging protocol

All patients underwent multidetector computed tomography (MDCT) of the brain, followed by CT angiography (CTA) for the evaluation of aneurysmal subarachnoid hemorrhage (aSAH). Standard protocols were followed for both non-contrast CT (NCCT) and contrast-enhanced CTA. Multiplanar reconstructions (MPR) in axial, coronal, and sagittal planes were performed to assess aneurysm characteristics, including size, shape, location, and multiplicity.

Imaging was performed using a 128-slice MDCT scanner (GE Revolution EVO, GE Medical Systems, Waukesha, WI). Each examination included both unenhanced (NCCT) and contrast-enhanced CTA phases. Patients were positioned supine with their arms by their sides. A scout view was obtained from the mid-chest to the vertex, and the scan was acquired from the aortic arch to the vertex in a craniocaudal direction during suspended respiration.

For CTA, a non-ionic contrast agent (120 mL; concentration exceeding 300 mg/mL) was administered via an 18-gauge catheter placed in the antecubital vein, using a power injector at a flow rate of 3-4 mL/s, followed by a 30 mL saline flush. Subsequent scan acquisitions were initiated at 20 and 60 seconds after the onset of contrast injection. Identical geometric settings were maintained across all scans to facilitate subtraction imaging. The imaging was performed using a 1-mm section thickness with a pitch range of 0.5-1. Scan parameters included a tube voltage of 120 kV, tube current of 300 mAs, a 200-mm field of view (FOV), and a 512 × 512 image matrix. Axial images were reconstructed in 5-mm contiguous slices. When necessary, subtracted images (pre- and post-contrast) and sagittal reformats were generated. The acquired axial images were transferred to a dedicated workstation for post-processing, where multiplanar reconstructions (MPR), three-dimensional CT angiography (3D-CTA), and maximum intensity projection (MIP) images were created. The 3D-CTA images were primarily generated using volume rendering techniques. To optimize vascular visualization, threshold values were typically set between 95 and 350 Hounsfield units (HU), with adjustments made based on the level of vascular enhancement. In cases of ruptured cerebral aneurysms, the hematoma was visualized with CT attenuation values ranging from approximately 75 to 90 HU, allowing clear distinction of vascular structures from subarachnoid hemorrhage.

Data collection

A structured case proforma was used to systematically collect data for each patient. This included demographic information such as age and gender, along with clinical history, findings from physical examination, and relevant blood biochemical parameters. Data collection was carried out after obtaining written informed consent from each participant, which included a detailed explanation of the procedure. Data collection was carried out by a junior radiologist (AT). CT angiographic data were independently interpreted by two radiologists: MS, with 25 years of experience, and PKS, with six years of experience. In cases of disagreement between their interpretations, a third senior radiologist (SP), with 35 years of experience, provided the final decision.

Aneurysm characteristics were meticulously documented, including size (measured in millimeters), shape (categorized as saccular or fusiform), location (e.g., anterior communicating artery, middle cerebral artery (MCA), or posterior circulation), and the presence of multiple aneurysms.

Statistical analysis

All categorical variables with adequate sample sizes were analyzed using the Chi-squared test. For variables with smaller sample sizes, regrouping was attempted based on methods adopted in previous similar studies. If regrouping was not feasible, Fisher's exact test was employed. The unpaired independent sample t-test was used to compare the mean age and aneurysm size between male and female patients. Proportions of categorical variables between genders were compared using the Z-test for proportions, assuming a normal distribution. A p-value of less than 0.05 was considered statistically significant (α = 0.05) for all statistical tests.

## Results

The study included 111 patients diagnosed with aneurysmal subarachnoid hemorrhage (aSAH), comprising 73 female patients and 38 male patients, with a female-to-male ratio of 1.92:1. Among male patients, 25 (65.79%) were below 55 years of age, while 13 (34.21%) were 55 years or older. In contrast, the majority of female patients (43 out of 73; 58.91%) were aged 55 years or above. This age distribution difference between genders was statistically significant (p = 0.0135). The mean age of female patients (56.11 years) was significantly higher than that of male patients (49.95 years), with a p-value of 0.020 (Table 1).

**Table 1:** Gender-based distribution of patient age *Chi-squared (χ²) test, †t-test SD: standard deviation

Age group (years)	Male (number (%))	Female (number (%))	Total (number (%))	p-value	Test statistic
<55	25 (65.8)	30 (41.1)	55 (49.5)	0.0135^*^	6.096
≥55	13 (34.2)	43 (58.9)	56 (50.5)		
Mean ± SD	49.95 ± 13.31	56.11 ± 12.51	54.00 ± 13.31	0.020^†^	-2.36

Gender-based differences in aneurysm multiplicity

**Figure 1:**
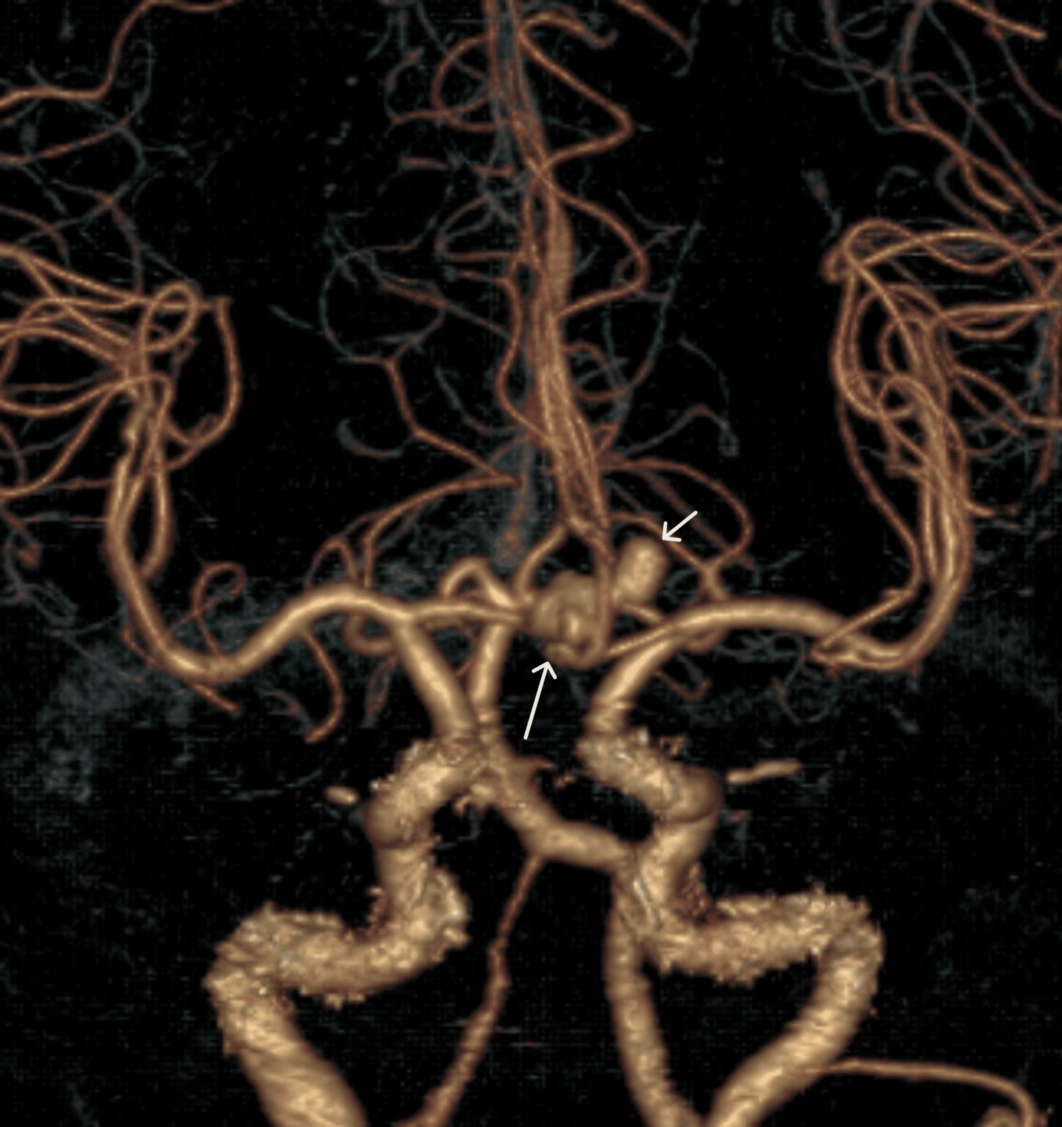
Axial CTA volume-rendered image demonstrating two aneurysms: one involving the ACOM (long white arrow) measuring 9.3 × 7.8 mm and the other involving the left ACA (short white arrow) measuring 5.8 × 5.5 mm CTA: computed tomography angiography, ACOM: anterior communicating artery, ACA: anterior cerebral artery

Out of the 111 patients, single aneurysms were observed in 95 (85.6%) cases, while multiple aneurysms were detected in 16 (14.4%) cases. Figure 1 shows two aneurysms involving the anterior communicating artery (ACOM) and the left anterior cerebral artery (ACA). 

Among male patients, two (5.3%) had double aneurysms, and 36 (94.7%) had single aneurysms. In female patients, 14 (19.18%) had double aneurysms, while 59 (80.82%) had single aneurysms. The difference in aneurysm multiplicity between genders was not quite statistically significant (p = 0.0518). The total number of aneurysms identified in the study was 127, comprising 16 cases of double aneurysms and 95 cases of single aneurysms. These results are presented in Table 2.

**Table 2:** Gender-based distribution of aneurysm multiplicity *Fisher's exact test

Multiplicity	Male (number (%))	Female (number (%))	Total (number (%))	p-value
Double aneurysms (yes)	2 (5.3)	14 (19.2)	16 (14.4)	0.0518*
Single aneurysm (no)	36 (94.7)	59 (80.8)	95 (85.6)	

Gender-based differences in aneurysm size

**Figure 2:**
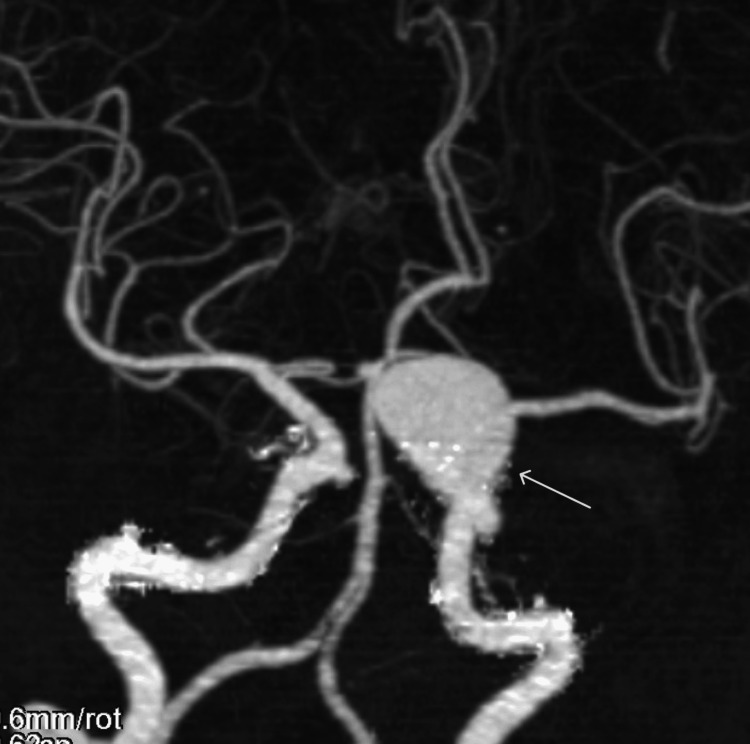
Coronal MIP CT angiographic image depicting a left ICA aneurysm (white arrow) MIP: maximum intensity projection, CT: computed tomography, ICA: internal carotid artery

A total of 127 aneurysms were analyzed. Of these, 77 (60.63%) aneurysms measured less than 6 mm, 36 (28.35%) were 6-10 mm, and 14 (11.02%) were 10-20 mm in size. No aneurysm larger than 20 mm was detected in this study. An example of a large left ICA aneurysm is shown in Figure 2.

Aneurysms measuring less than 6 mm were more frequently observed in female patients (66.67%) than in male patients (47.5%), and this difference was statistically significant (p = 0.0404). Aneurysms in the 6-10 mm size range were more common among male patients (32.5%) than female patients (26.43%), but the difference was not statistically significant (p = 0.484). Aneurysms between 10 and 20 mm were more common in male patients (20%) compared to female patients (6.9%), with a statistically significant difference (p = 0.0285).

The mean size of aneurysms in male patients was 7.29 mm, which was significantly larger than that in female patients (6.15 mm), with a p-value of 0.026. These results are presented in Table 3.

**Table 3:** Gender-based distribution of aneurysm size *Z-test, †t-test SD: standard deviation

Aneurysm size (mm)	Male (number (%))	Female (number (%))	Total (number (%))	p-value	Test statistic
<6	19 (47.5)	58 (66.7)	77 (60.6)	0.0404^*^	-2.05
6-10	13 (32.5)	23 (26.4)	36 (28.3)	0.484^*^	0.704
10-20	8 (20.0)	6 (6.9)	14 (11.0)	0.0285^*^	2.190
>20	0 (0.0)	0 (0.0)	0 (0.0)	-	-
Mean ± SD	7.29 ± 2.67	6.15 ± 2.49	6.51 ± 2.60	0.026^†^	2.262

Gender-based differences in aneurysm shape

**Figure 3:**
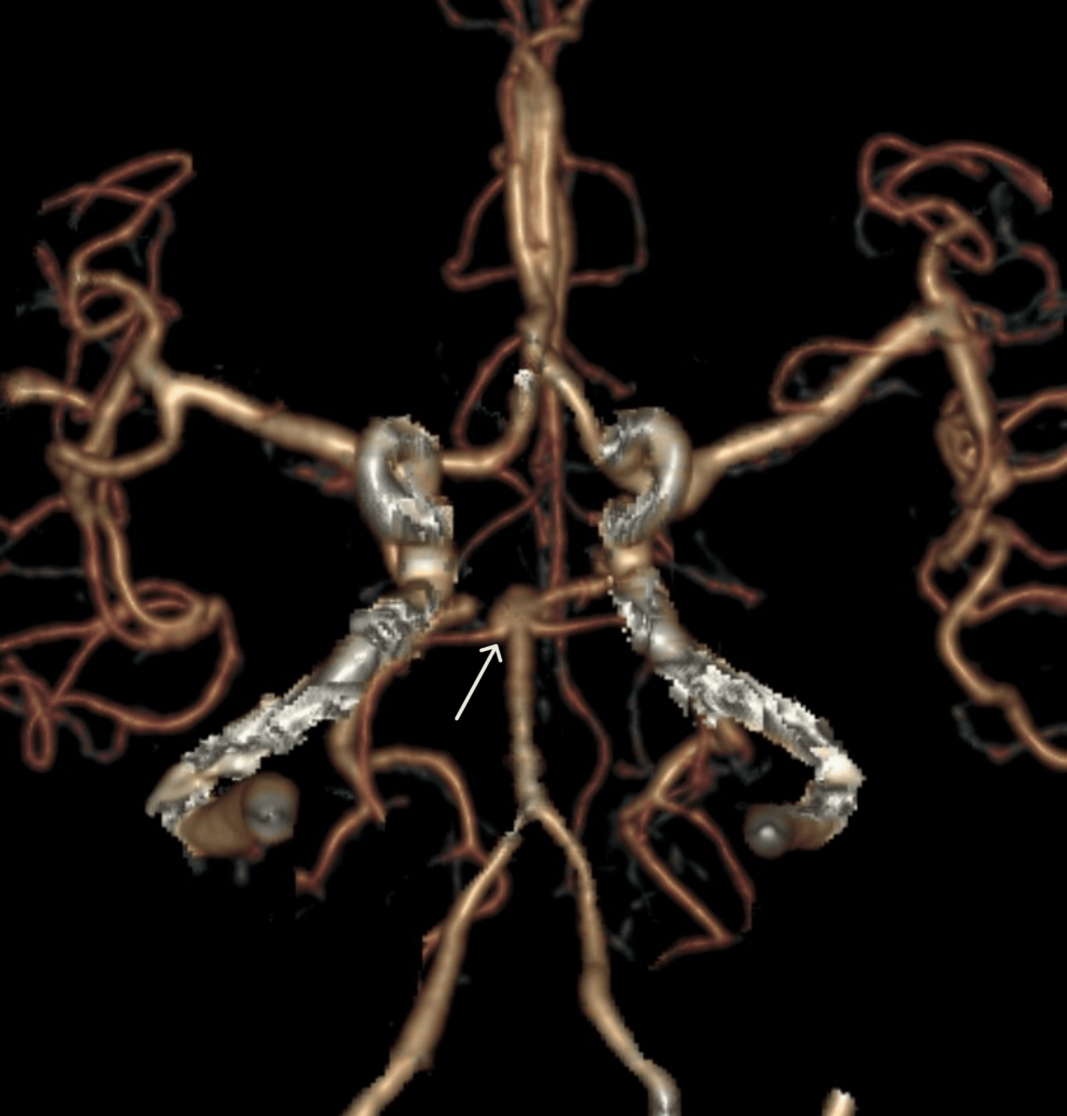
Coronal CT angiography VR image demonstrating a fusiform aneurysm at the top of the basilar artery (white arrow) CT: computed tomography, VR: volume rendering

Among the 127 aneurysms identified, 118 (92.9%) were saccular, while nine (7.1%) were fusiform in shape (Figure 3). Of the 40 aneurysms seen in male patients, 34 (85%) were saccular and six (15%) were fusiform. In contrast, of the 87 aneurysms in female patients, 84 (96.5%) were saccular and only three (3.5%) were fusiform. Fusiform aneurysms showed a male preponderance, and this gender-based difference was statistically significant (p = 0.0273).

Among the nine fusiform aneurysms, six (66.67%) were located in the vertebrobasilar artery (VBA) territory, and three (33.33%) were found in the middle cerebral artery (MCA) territory. These results are presented in Table 4.

**Table 4:** Gender-based distribution of aneurysm shape *Fisher's exact test

Shape	Male (number (%))	Female (number (%))	Total (number (%))	p-value
Saccular	34 (85.0)	84 (96.5)	118 (92.9)	0.0273^*^
Fusiform	6 (15.0)	3 (3.5)	9 (7.1)	

Gender-based differences in aneurysm location

Among the 127 aneurysms identified, 38 (29.92%) were located in the anterior cerebral artery (ACA) territory, 30 (23.62%) in the middle cerebral artery (MCA) territory, 49 (38.58%) in the internal carotid artery (ICA) territory, and 10 (7.88%) in the vertebrobasilar artery (VBA) territory, as shown in Table 5. Overall, aneurysms were predominantly located in the anterior circulation (117, 92.12%) compared to the posterior circulation (10, 7.88%).

**Table 5:** Gender-based distribution of aneurysm location *Z-test ACOM: anterior communicating artery, PCOM: posterior communicating artery, BA: basilar artery, PCA: posterior cerebral artery, VA: vertebral artery, ACA: anterior cerebral artery, MCA: middle cerebral artery, ICA: internal carotid artery, VBA: vertebrobasilar artery

Vascular territory	Location	Female (number (%))	Male (number (%))	Total (number (%))	p-value	Test statistic (Z score)
ACA	A1 segment	3 (3.45)	0 (0.0)	3 (2.36)	-	-
	ACOM	16 (18.39)	15 (37.5)	31 (24.41)	0.0198	-2.329
	A2 segment	2 (2.30)	2 (5.0)	4 (3.15)	-	-
	Subtotal	21 (24.14)	17 (42.5)	38 (29.92)	0.0357	-2.099
MCA	M1 segment	14 (16.09)	6 (15.0)	20 (15.75)	0.8729	0.157
	M2 segment	6 (6.90)	4 (10.0)	10 (7.87)	-	-
	Subtotal	20 (22.99)	10 (25.0)	30 (23.62)	0.8026	-0.248
ICA	ICA	24 (27.58)	5 (12.5)	29 (22.83)	-	-
	PCOM	18 (20.69)	2 (5.0)	20 (15.75)	-	-
	Subtotal	42 (48.27)	7 (17.5)	49 (38.58)	<0.001	3.309
VBA	BA	2 (2.30)	4 (10.0)	6 (4.72)	-	-
	PCA	1 (1.15)	2 (5.0)	3 (2.36)	-	-
	VA	1 (1.15)	0 (0.0)	1 (0.79)	-	-
	Subtotal	4 (4.60)	6 (15.0)	10 (7.88)	-	-

Of the 40 aneurysms observed in male patients, 17 (42.5%) were in the ACA territory, 10 (25%) in the MCA, seven (17.5%) in the ICA, and six (15%) in the VBA territory. In contrast, of the 87 aneurysms seen in female patients, 21 (24.14%) were in the ACA territory, 20 (22.99%) in the MCA, 42 (48.27%) in the ICA, and four (4.6%) in the VBA territory. ACA territory aneurysms were significantly more common in male patients (42.5%) than in female patients (24.14%), while ICA aneurysms were significantly more common in female patients (48.27%) than in male patients (17.5%) (p < 0.001).

**Figure 4:**
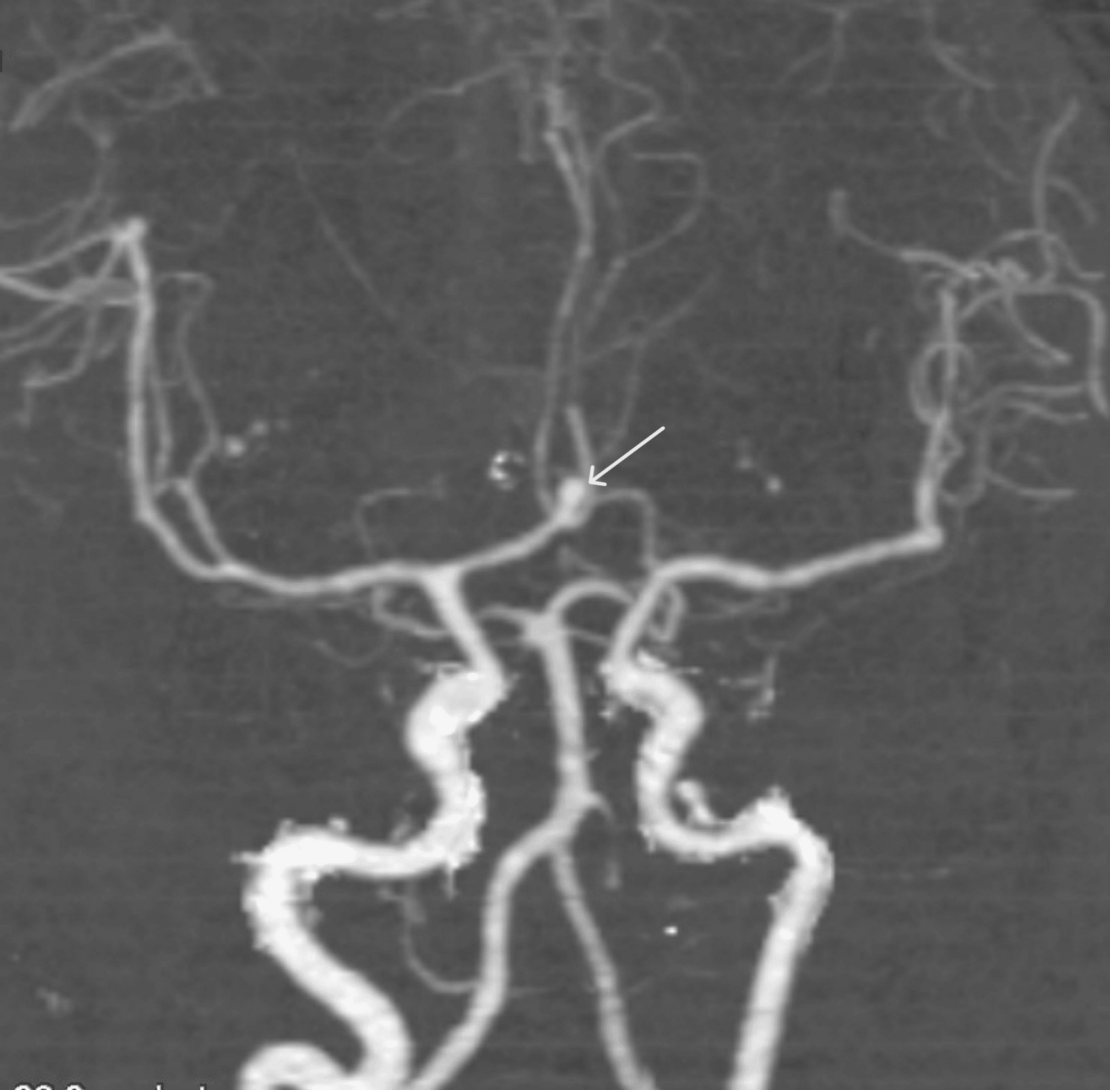
Axial MIP CT angiography image showing a saccular aneurysm at the ACOM (white arrow) MIP: maximum intensity projection, CT: computed tomography, ACOM: anterior communicating artery

When further subcategorized, the most common aneurysm location in the overall study population was the anterior communicating artery (ACOM) at 24.41% (Figure 4), followed closely by the MCA at 23.62% and ICA at 22.83%. Among male patients, ACOM aneurysms were most common (37.5%; p = 0.0198), whereas in female patients, ICA aneurysms were more frequent (27.58%). However, a p-value could not be calculated for the comparison of ICA aneurysms between males and females because the number of males with ICA aneurysms was too small to allow for a valid statistical analysis.

## Discussion

Among the 111 patients with aneurysmal subarachnoid hemorrhage (aSAH), there was a clear female preponderance, with a female-to-male ratio of 1.92:1. Most male patients were under 55 years of age, with a mean age of 49.95 years. In contrast, most female patients were over 55 years, with a mean age of 56.11 years, suggesting that females with aSAH tend to be older than their male counterparts.

This observation aligns with the findings of Hamdan et al., who reported that female patients were significantly older than male patients (mean age: 56.6 versus 51.9 years), with a higher proportion of women aged ≥55 years (56.2% versus 40.4%) [8]. Similarly, Kongable et al. found that women in their study were older than men (mean age: 51.4 versus 47.3 years; p < 0.001) [9]. Supporting our results, Kaminogo et al. also observed that women aged ≥50 years outnumbered all other age and sex categories [10].

Multiple aneurysms were more frequently observed in female patients (19.18%) compared to male patients (5.3%). Overall, 14.44% of patients had multiple aneurysms, with the internal carotid artery (ICA) being the most common site of multiple aneurysms among female patients (50%).

Our findings are consistent with those of Hamdan et al., who reported a higher prevalence of multiple aneurysms in female patients than in male patients (11.5% versus 5.2%) [8]. Similarly, Ghods et al. also found that multiple aneurysms were more common in female patients (11%) compared to male patients (6%) [11].

Most of the aneurysms measured less than 6 mm (60.63%), followed by those in the 6-10 mm category (28.35%). Aneurysms smaller than 6 mm were more common in female patients (66.67%) compared to male patients (47.5%). Conversely, aneurysms larger than 10 mm were more frequently observed in male patients (20%) than in female patients (6.9%). The mean aneurysm size was also larger in male patients (7.29 mm) than in female patients (6.15 mm).

In contrast to our findings, Ghods et al. reported no significant difference in the mean size of ruptured aneurysms between genders (5.9 mm versus 6.1 mm) [11]. However, Lin et al. found, similar to our study, that aneurysm sizes (p = 0.001) were significantly larger in male patients, particularly in ruptured anterior communicating artery (ACOM) aneurysms [12]. On the other hand, Imaizumi et al. reported that the majority of aneurysms were in the 2.0-2.9 mm range [13]. This discrepancy may be attributed to the use of magnetic resonance angiography (MRA) in their study, which focused on detecting unruptured aneurysms.

Among the 127 aneurysms in our cohort, saccular aneurysms were predominant, accounting for 118 (92.9%) cases, while fusiform aneurysms were observed in only nine (7.1%) cases. Our findings align with those of Gasparotti et al., who reported that saccular aneurysms constitute approximately 90% of all intracranial aneurysms [14].

Of the 40 aneurysms observed in male patients, 34 (85%) were saccular and six (15%) were fusiform. In female patients, 84 out of 87 aneurysms (96.5%) were saccular and only three (3.5%) were fusiform. A significant male preponderance was noted in fusiform aneurysms (p = 0.0273, statistically significant at α = 0.05). Among the nine fusiform aneurysms, six (66.67%) were located in the vertebrobasilar (VBA) territory and three (33.33%) in the middle cerebral artery (MCA) territory.

These observations are consistent with the findings of Park et al., who described intracranial fusiform aneurysms as rare, accounting for approximately 3%-13% of all intracranial aneurysms [15]. They are predominantly located in the vertebrobasilar system and are more commonly seen in younger patients and males.

Anterior circulation aneurysms were significantly more common (92.2%) than those in the posterior circulation (7.8%). The most frequent aneurysm location was the internal carotid artery (ICA) (38.58%), followed by the anterior cerebral artery (ACA) (29.92%) and MCA (23.62%). Female patients showed a higher prevalence of ICA aneurysms (48.27%), whereas ACA aneurysms were more common in male patients (42.5%). Upon further subcategorization, ACOM aneurysms were most frequent in male patients (37.5%), while ICA aneurysms were predominant in female patients (27.58%).

Among the 127 aneurysms, 38 (29.92%) were located in the anterior cerebral artery (ACA) territory, 30 (23.62%) in the middle cerebral artery (MCA) territory, 49 (38.58%) in the internal carotid artery (ICA) territory, and 10 (7.88%) in the vertebrobasilar (VBA) territory. These findings suggest a greater involvement of the ICA territory, followed by the ACA, in cases of aneurysmal subarachnoid hemorrhage (SAH), compared to the MCA and VBA territories. Overall, there was a marked predominance of aneurysms in the anterior circulation (117 cases, 92.22%) compared to the posterior circulation (10 cases, 7.88%). These results are consistent with those of Gasparotti et al., who reported posterior circulation aneurysms in 8%-10% of cases [14].

Among the 40 aneurysms in male patients, 17 (42.5%) were in the ACA territory, 10 (25%) in the MCA, seven (17.5%) in the ICA, and six (15%) in the VBA territory. Of the 87 aneurysms in female patients, 21 (24.14%) were in the ACA territory, 20 (22.99%) in the MCA, 42 (48.27%) in the ICA, and four (4.6%) in the VBA territory. ACA territory aneurysms were significantly more common in male patients (42.5%) compared to female patients(24.14%), whereas ICA territory aneurysms were more common in female patients (48.27%) than in male patients (17.5%) (p < 0.001, statistically significant at α = 0.05).

Our findings are in line with those of Kongable et al., who reported that female patients more frequently harbored ICA aneurysms than male patients (36.8% versus 18.0%; p < 0.001) and were also more likely to have multiple aneurysms (32.4% versus 17.6%; p < 0.001) [9]. Conversely, ACA aneurysms were more commonly encountered in men (46.1%) than in women (26.6%; p < 0.001). Similarly, Lindner et al. observed a higher incidence of ICA aneurysms in women and ACA aneurysms in men [16].

Upon subcategorization, anterior communicating artery (ACOM) aneurysms were the most common overall (24.41%), followed closely by MCA (23.62%) and ICA (22.83%) aneurysms. Among male patients, aneurysms involving the anterior communicating artery (ACOM) were the most common (37.5%), with the association being statistically significant (p = 0.0198). In contrast, among female patients, internal carotid artery (ICA) aneurysms were more frequent (27.58%). Kaminogo et al. similarly found that in SAH patients with a single aneurysm, the most frequent site in men was ACOM (41.2%), whereas in women, it was the ICA (32.0%) [10]. Hamdan et al. also observed ACOM as the predominant aneurysm site in men (42.3%) [8]. However, in contrast to our findings, they reported that in women, posterior communicating artery (PCOM) aneurysms (27.3%) were the most common.

While this study provides valuable insights into gender-based differences and aneurysm characteristics in aneurysmal subarachnoid hemorrhage (aSAH), several limitations should be acknowledged. First, a key limitation of our study is the unavailability of digital subtraction angiography (DSA) at our institution during the study period, which precluded direct comparison with CT angiography, despite DSA's established superiority in detecting small aneurysms (<3 mm). Second, the study was carried out at a single tertiary care center, which may restrict the applicability of its findings to wider or more diverse populations. Third, although the sample size was adequate for preliminary observations, it may not be sufficient to draw definitive conclusions for all subgroups, such as those with rare aneurysm locations or giant aneurysms. Fourth, the retrospective nature of image interpretation may introduce observer bias, despite efforts to standardize reporting protocols. Additionally, follow-up clinical outcomes were not assessed, restricting the ability to correlate imaging findings with patient prognosis. Moreover, due to the unavailability of digital subtraction angiography (DSA), correlation with this gold standard modality could not be performed. Future multicentric studies with larger cohorts and long-term outcome analysis are recommended to validate and build upon these findings.

## Conclusions

Multidetector computed tomography (MDCT) with CT angiography is a fast, non-invasive imaging modality that effectively identifies aneurysms in patients with aneurysmal subarachnoid hemorrhage (aSAH).

There is a higher prevalence of aSAH among female patients. Female patients are generally older, have a greater tendency to develop multiple aneurysms, and more frequently harbor internal carotid artery (ICA) aneurysms. In contrast, male patients are typically younger and more commonly present with anterior cerebral artery (ACA) aneurysms, particularly those involving the anterior communicating artery (ACOM). Most aneurysms were located in the anterior circulation and were saccular in morphology. Fusiform aneurysms were more common in male patients and predominantly found in the vertebrobasilar circulation. Most aneurysms were smaller than 6 mm. Although aneurysms were more commonly observed in female patients, male patients demonstrated a statistically significantly larger mean aneurysm size compared to female patients.
